# Transcriptome profiling and digital gene expression analysis of sweet potato for the identification of putative genes involved in the defense response against *Fusarium oxysporum* f. sp. *batatas*

**DOI:** 10.1371/journal.pone.0187838

**Published:** 2017-11-13

**Authors:** Yuli Lin, Weikun Zou, Shiqiang Lin, Dennis Onofua, Zhijian Yang, Haizhou Chen, Songliang Wang, Xuanyang Chen

**Affiliations:** 1 Key Laboratory of Crop Biotechnology (Fujian Agriculture and Forestry University), Fujian Province University, Fuzhou, Fujian, China; 2 Key Lab of Genetics, Breeding and Multiple Application of Crops (FAFU), Ministry of Education, Fuzhou, Fujian, China; 3 Department of Agronomy, College of Crop Science, Fujian Agriculture and Forestry University, Fuzhou, Fujian, China; 4 Department of Bioinformatics, College of Life Science, Fujian Agriculture and Forestry University, Fuzhou, Fujian, China; 5 Fujian Provincial Key Laboratory of Crop Breeding by Design, Fuzhou, Fujian, China; Bhabha Atomic Research Centre, INDIA

## Abstract

Sweet potato production is constrained by Fusarium wilt, which is caused by *Fusarium oxysporum* f. sp. *batatas (*Fob). The identification of genes related to disease resistance and the underlying mechanisms will contribute to improving disease resistance via sweet potato breeding programs. In the present study, we performed de novo transcriptome assembly and digital gene expression (DGE) profiling of sweet potato challenged with Fob using Illumina HiSeq technology. In total, 89,944,188 clean reads were generated from 12 samples and assembled into 101,988 unigenes with an average length of 666 bp; of these unigenes, 62,605 (61.38%) were functionally annotated in the NCBI non-redundant protein database by BLASTX with a cutoff E-value of 10^−5^. Clusters of Orthologous Groups (COG), Gene Ontology (GO) and Kyoto Encyclopedia of Genes and Genomes (KEGG) annotations were examined to explore the unigenes’ functions. We constructed four DGE libraries for the sweet potato cultivars JinShan57 (JS57, highly resistant) and XinZhongHua (XZH, highly susceptible), which were challenged with pathogenic Fob. Genes that were differentially expressed in the four libraries were identified by comparing the transcriptomes. Various genes that were differentially expressed during defense, including chitin elicitor receptor kinase 1 (CERK), mitogen-activated protein kinase (MAPK), WRKY, NAC, MYB, and ethylene-responsive transcription factor (ERF), as well as resistance genes, pathogenesis-related genes, and genes involved in salicylic acid (SA) and jasmonic acid (JA) signaling pathways, were identified. These data represent a sequence resource for genetic and genomic studies of sweet potato that will enhance the understanding of the mechanism of disease resistance.

## Introduction

Sweet potato is the 7th most important crop globally, with an annual area harvest of 8.0 million ha and a total global production of 104.5 million tons. Sweet potato is planted primarily in developing areas in Asia and Africa [1, 2]. In the past two decades, sweet potato production has decreased in Asia and greatly increased in Africa [2]. In the USA, the harvest area has increased from 33.3 kha in 2002 to 54.7 kha in 2014, and this increase has resulted in a corresponding increase in sweet potato production from 580.5 kilotons to 1.34 million tons [2].

*Fusarium oxysporum*, a soil-borne pathogenic fungus, is classified into more than 120 *formae speciales* and races, each of which infects one specific crop, such as sweet potato, tomato, banana, watermelon or pepper. The severity of the damage caused by these infections depends on the interaction between the pathogen and the host crop [3]. The production of sweet potato is constrained by Fusarium wilt, which is caused by *Fusarium oxysporum* f. sp. *batatas* (Fob) [4]. Sweet potato growth was limited in the Southeastern United States prior to the 1950s and South China prior to the 1980s, when resistant cultivars were bred and applied [5, 6]. Fusarium wilt has spread across many provinces in Central China and threatens sweet potato production [7].

During Fob infections, sweet potato secretes tyloses, cell wall material or brown matter into xylem vessels as a physiological response to impede Fob infection, leading to blockade of the vascular bundle [8, 9] and stem split and rot [5]. Colonization by Fob can induce the defense system in sweet potato. The susceptibility or resistance of sweet potato to Fusarium wilt depends on the interaction with Fob, consistent with the gene-for-gene hypothesis proposed by Flor [10] and the mode of co-evolution proposed by Jones [11]. The pathogen-associated molecular patterns (PAMPs), such as chitin, glucan, or glycoprotein, of fungal cells are recognized by pattern recognition receptors (PRRs) in plants, resulting in PAMP-triggered immunity (PTI) to prevent pathogen colonization. Successful pathogens release effector proteins to suppress PTI [11]. In response, plants have evolved resistance (R) proteins to recognize these effectors and trigger a secondary response, i.e., effector-triggered immunity (ETI), which induces the expression of downstream defense-related genes [12]. This model was elucidated based on the interaction between tomato and its pathogenic *Fusarium oxysporum* f. sp. *lycopersici* (Fol) [13]. Many R genes with identified functions have been isolated from Arabidopsis, rice, tomato, and other host plants [14–17].

Sweet potato is hexaploid with a complex genome that has not been sequenced [18]. High-throughput RNA sequencing based on next-generation sequencing technology (Illumina) has recently been applied in analyses of gene expression profiling and for the discovery of important genes involved in disease resistance and related pathways [19, 20]. Transcriptome sequencing offers a rapid approach to obtain expressed gene sequence information for sweet potato [21] and has been used to mine gene-based microsatellite markers [22, 23], elucidate the mechanism of development and metabolism in the purple tuberous roots of sweet potato [18], reveal gene expression patterns associated with tuberous root formation and development [24], and identify related genes involved in lignin and starch biosynthesis [21].

In the present study, transcriptome sequencing technology was used to perform RNA sequencing (RNA-Seq) of sweet potato cultivars inoculated with pathogenic *F*. *oxysporum*. We constructed a sufficiently large library using equally mixed total RNA. In total, 89,944,188 high-quality reads were retained and assembled into 62,605 unigenes. The unigenes were then annotated and assigned putative functions, classifications or pathways by alignment with public databases. The transcriptome data provide a global view of the gene expression in sweet potato during Fob infection, generating a resource for discovering candidate defense-related genes. Based on the transcriptome data, we constructed cDNA libraries and performed four digital gene expression (DGE) analyses using sequencing reads from the libraries to compare the gene expression profiles of a sweet potato cultivar challenged with pathogenic Fob and identify a set of genes involved in the defense response to Fob. The comparisons will provide a better understanding of the molecular mechanisms underlying the defense response of sweet potato to Fob.

## Materials and methods

### Plant and pathogen materials, treatment and RNA extraction

Two sweet potato cultivars were used as the plant materials: Jinshan57 (JS57, highly resistant to Fusarium wilt) and XinZhongHua (XZH, highly susceptible to Fusarium wilt). JS57 was used as a highly resistant control (disease index of 14.7), whereas XZH was used as a highly susceptible control (disease index of 100) to identify resistance to Fusarium wilt [4].

The virulent pathogen fungus *Fusarium oxysporum* f. sp. *batatas*07 (F07) was used. F07 was isolated from sweet potato with Fusarium wilt and stored at -20°C.

Seedlings of sweet potato were inoculated with F07 or water (as a control). Four samples were prepared as follows: (1) JS57 inoculated with F07 (JS57-F07); (2) JS57 in water (JS57-CK); (3) XZH inoculated with F07 (XZH-F07); and (4) XZH in water (XZH-CK).

The conidia solutions were prepared as follows: F07 was grown on potato sugar agar (PSA) medium in a Petri dish for 8 d at 28°C until the mycelia grew over the surface of the medium. The PSA medium with mycelia was cut into small pieces and transferred into 250-mL conical flasks containing 100 mL of liquid Czapek medium. Each flask containing eight pieces of PSA medium with mycelia was incubated at 28°C for 8 d on a rotary shaker. The liquid medium containing the conidia and mycelia was strained through cheesecloth, and the conidia were collected into clean empty flasks. The conidia concentration of the solution in the flask was determined using a hemocytometer. Prior to incubation, the conidia suspensions were diluted in sterilized distilled water to a concentration of 1 × 10^7^ conidia/mL.

Healthy sweet potato seedlings that were field grown for at least 28 d and had no obvious disease symptoms were freshly cut to a length of 20 cm and cultivated in glass bottles containing conidial solutions of 1 × 10^7^ conidia/mL. Seedlings were cultivated in water as a control. The seedlings were cultivated in growth chambers at 28°C for 24 h, and the basic stems of seedlings with a length of 2 cm were collected as samples. Three independent biological replicates of each treatment were collected and immediately frozen in liquid nitrogen. Total RNA was isolated from each sample using the RNAprepPure Plant Kit (Tianen Biotech, Beijing, China), according to the manufacturer's instructions. RNA degradation was detected by electrophoresis on 1% agarose gels, and the RNA purity was measured by a NanoPhotometer spectrophotometer (IMPLEN, CA, USA). We used the Qubit RNA Assay Kit with a Qubit2.0 Fluorometer (Life Technologies, CA, USA) to determine the RNA concentration. The RNA integrity was confirmed using an RNA Nano 6000 Assay Kit on the Agilent Bioanalyzer 2100 system (Agilent Technologies, CA, USA).

### Library preparation and transcriptome sequencing

The sequencing library was prepared using 3 μg of RNA from each sample and the NEBNextUltra™ RNA Library Prep Kit for Illumina (NEB, USA) according to the manual. Index codes were added to sequences from each sample. In brief, poly-T oligo-attached magnetic beads were applied to purify mRNA from total RNA. Then, fragmentation was conducted in the presence of divalent cations and at higher temperature in NEBNext, First Strand Synthesis Reaction Buffer (5X). We utilized random hexamer primers and M-MuLV Reverse Transcriptase to perform first-strand cDNA synthesis and DNA Polymerase I and RNase H for the second-strand cDNA synthesis. The overhangs were filled to blunt ends by exonuclease/polymerase activity. Then, the 3’ ends of the DNA fragments were adenylated, and NEBNext, Adaptors with hairpin loop structures were ligated to the DNA fragments. cDNA fragments with a size of 150–200 bp were purified with the AMPure XP system (Beckman Coulter, Beverly, USA). The cDNA was then incubated with the USER Enzyme (NEB, USA) at 37°C for 15 min and 95°C for 5min. PCR was performed using Phusion High-Fidelity DNA polymerase with Universal PCR primers and Index (X) Primer. The PCR products were purified with an AMPure XP system. The library quality was evaluated with the Agilent Bioanalyzer 2100 system. The index-coded samples were clustered using the cBot Cluster Generation System with the TruSeq PE Cluster Kit v3-cBot-HS (Illumina) according to the manual. The library preparations were then sequenced on an Illumina HiSeq 2500 platform. The resultant datasets are available in the NCBI Gene Expression Omnibus repository under GEO Series accession number GSE89290 (https://www.ncbi.nlm.nih.gov/geo/query/acc.cgi?acc=GSE89290).

### Quality control and de novo assembly

The data produced using the Illumina HiSeq 2500 platform were transferred into raw reads (raw data) using base calling. The raw reads were initially processed via in-house Perl scripts [25], and the reads containing adapters or N (indefinite base) higher than 10%as well as low-quality reads were then removed. The remaining high-quality clean reads were used to determine the error rate, Q20, Q30, GC content and sequence duplication level. The clean data were also used to perform the following bioinformatics analyses. According to Haas et al. [25], the clean reads were concatenated into two files. The left files (read1 files) from all libraries/samples were pooled into a large left.fq file, and the right files (read2 files) were pooled into a large right.fq file. The transcriptome assembly was obtained based on the left.fq and right.fq using Trinity [26] for de novo assembly, with a minimum kmer coverage of two and default setting for all other parameters.

### Gene functional annotation

The functions of the assembled transcripts were annotated by searching their sequences against the following databases:

NR (NCBI non-redundant protein sequences, http://www.ncbi.nlm.nih.gov) with an E-value cutoff of 1e-5;

Nt (NCBI non-redundant nucleotide sequences, http://www.ncbi.nlm.nih.gov) with an E-value cutoff of 1e-5;

PFAM (Protein family, http://pfam.sanger.ac.uk/) with an E-value cutoff of 1e-2;

KOG/COG (Clusters of Orthologous Groups of proteins, http://www.ncbi.nlm.nih.gov/COG/) with an E-value cutoff of 1e-3; and

Swiss-Prot (A manually annotated and reviewed protein sequence database, http://www.ebi.ac.uk/uniprot/) with an E-value cutoff of 1e-5.

Based on the annotations in NR and PFAM, Blast2GO (v2.5) was used to obtain the GO annotations (http://www.geneontology.org) according to the molecular function, biological process and cellular component ontologies [27]. The Automatic Annotation Server and the Kyoto Encyclopedia of Genes and Genomes (KEGG) pathway database (http://www.genome.jp/kegg) were used to generate pathway assignments with an E-value cutoff of 1e-5.

### Differential expression analysis

The analysis of differential expression between the four groups was performed with the DESeq R package (1.10.1). The resulting P-values were adjusted according to Benjamini and Hochberg’s approach for controlling the false discovery rate. Genes with an adjusted P-value <0.05, as determined by DESeq, were regarded as differentially expressed.

### Experimental validation

Five genes were examined using real-time quantitative PCR (Q-PCR) to confirm their expression levels using FPKM, and the primers (Table 1) were designed using software Primer 5 and synthesized by Invitrogen. The primer sequences are listed in Table 1. A total of 1 μg of total RNA was used to synthesize the cDNA using the Prime Script RT Reagent Kit (Takara, DaLian, China) in a 20-μL reaction mixture. qRT-PCR was performed on a StepOnePlus™ Real-Time PCR System (Thermo Fisher Scientific), and the sweet potato GAPDH gene [28] was used as the endogenous reference control. All reference and selected genes were measured in triplicate.

**Table 1:** Sequences of primers used in the qRT-PCR analysis.

Sample	Gene ID	Primers	Primer sequences
JS57	C52297	F	CCCTTCATTCCCTTTCCACC
		R	CGAGTTCTTCGTCGCCGTTA
	C51550	F	TTTGTTTCTTCCACCTCTGT
		R	ATGCACGAATATCGCCTTAG
	C47391	F	TGTTGGGTCTGATAGTATTG
		R	CTCTTCCCTTTATTCTTCTG
XZH	C55420	F	CAGCCCAATAATCCAAGCAC
		R	GCATCACATCGGACCCTAAC
	C56184	F	TACCAATCAAAGGCTCACAA
		R	CCACCTAAAGGCACCAAGAC
Reference	GAPDH	F	GCAGGAACCCGGAAGAGATT
		R	GCAGCCTTGTCCTTGTCAGTG

Each reaction had a total volume of 20 μL and contained 2 μL of cDNA, 0.2 μL (10 μmol L^-1^) of gene-specific primers and 0.1 μL of CXR SYBR Green Master Mix. The real-time quantitative PCR program was 95°C for 10 min followed by 40 cycles of95°C for 15 s, 55°C for 20 s and 72°C for 30 s. The data were processed with Excel 2007(Microsoft, WA, USA).

## Results

### RNA-Seq and de novo transcriptome assembly

To comprehensively generate the transcriptome and obtain insights into the molecular mechanisms involved in the resistance of sweet potato to Fusarium wilt, the total RNA from the stems of the highly resistant cultivar JS57 and highly susceptible cultivar XZH was isolated. The RNAs were equally mixed to create an RNA pool for the cDNA library, and a database of the sweet potato stem transcriptome was then generated through Illumina HiSeq^TM^ 2500 sequencing. The deep sequencing produced 91,366,006 sequence reads with a length of 100 bp each and an average GC content of 45.10%. After removing adaptors, primer sequences, poly-A tails and short and low-quality sequences, 89,944,188 (98.44%, 11.24 Gbp) high-quality reads with Q≥20 were retained and used for the assembly.

**Fig 1:**
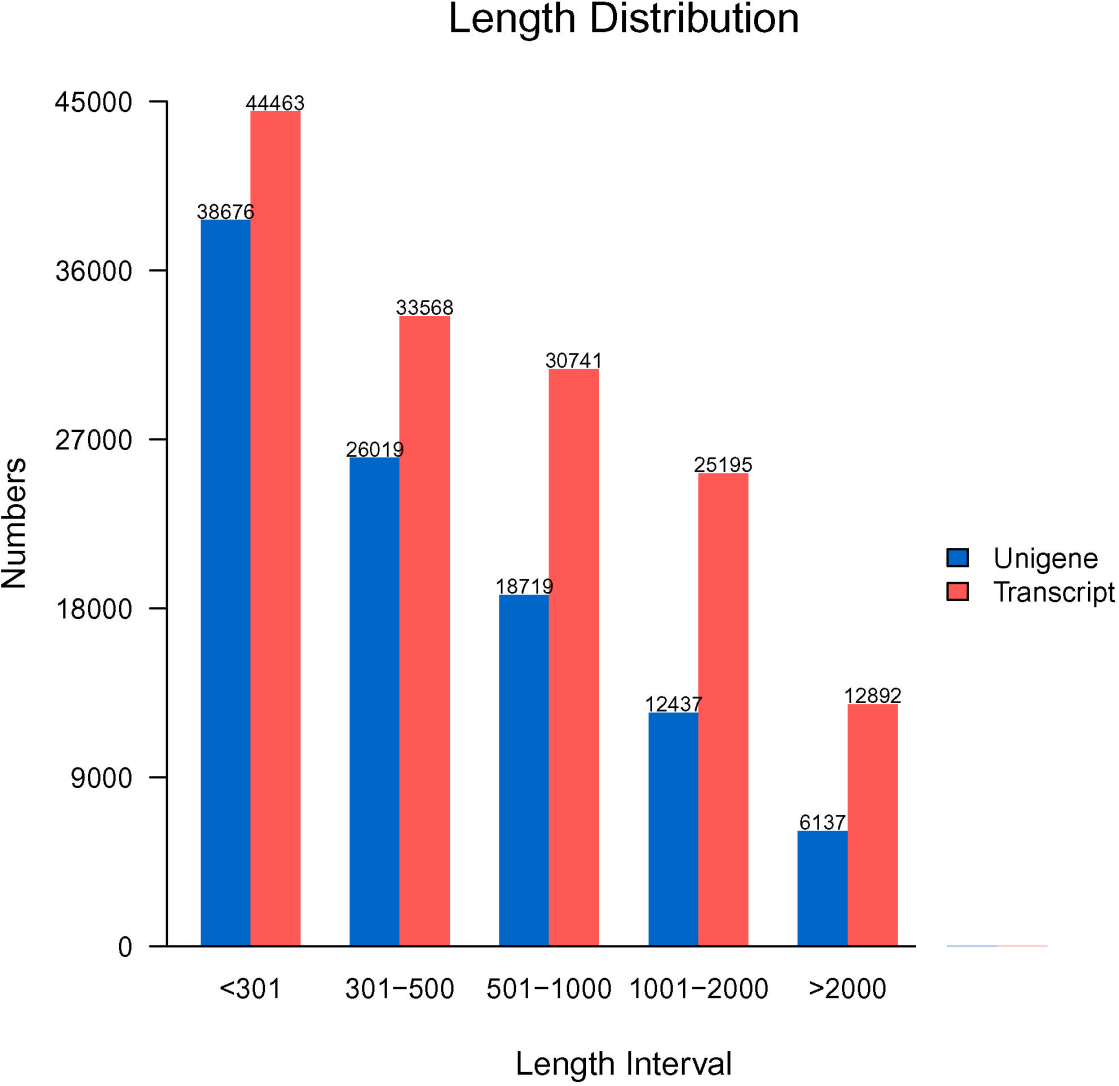
Length distribution of the assembled transcripts and unigenes in sweet potato. The horizontal axis represents the length intervals of the transcripts and unigenes, and the vertical axis represents the number of transcripts and unigenes.

Due to the absence of a sequenced genome, all trimmed and cleaned reads were aligned and assembled using Trinity [29], yielding a set of 146,859 transcript sequences that were longer than 201 bp with an N50 length of 1371 bp and 101,988 unigenes with an average length of 666 bp and an N50 length of 1079 bp. The length of the assembled unigenes varied from 201 to 15,576 bp, and more than 36.6% of these unigenes were longer than 501 bp, consistent with previously reported transcriptome data from sweet potato (Tables 2 and 3, Fig 1) [24, 30, 31].

**Table 2:** Frequency of the length distribution of the assembled transcripts and unigenes.

Transcript length interval	200–500 bp	500–1 kb	1–2 kb	>2 kb	Total
Number of transcripts	78031	30741	25195	12892	146859
Number of unigenes	64695	18719	12437	6137	101988

**Table 3:** Length distribution of the assembled transcripts and unigenes.

	Min Length	Mean Length	Median Length	Max Length	N50	N90	Total Nucleotides
Transcripts	201	812	459	15576	1371	314	119187574
Unigenes	201	666	367	15576	1079	267	67942174

The lack of a reference sweet potato genome sequence is a barrier to identifying candidate genes involved in disease resistance. The overview of the gene expression profile of sweet potato challenged with Fob obtained in this study offers abundant sequence data for identifying candidate genes associated with defense against Fusarium wilt.

### Functional annotation

**Fig 2:**
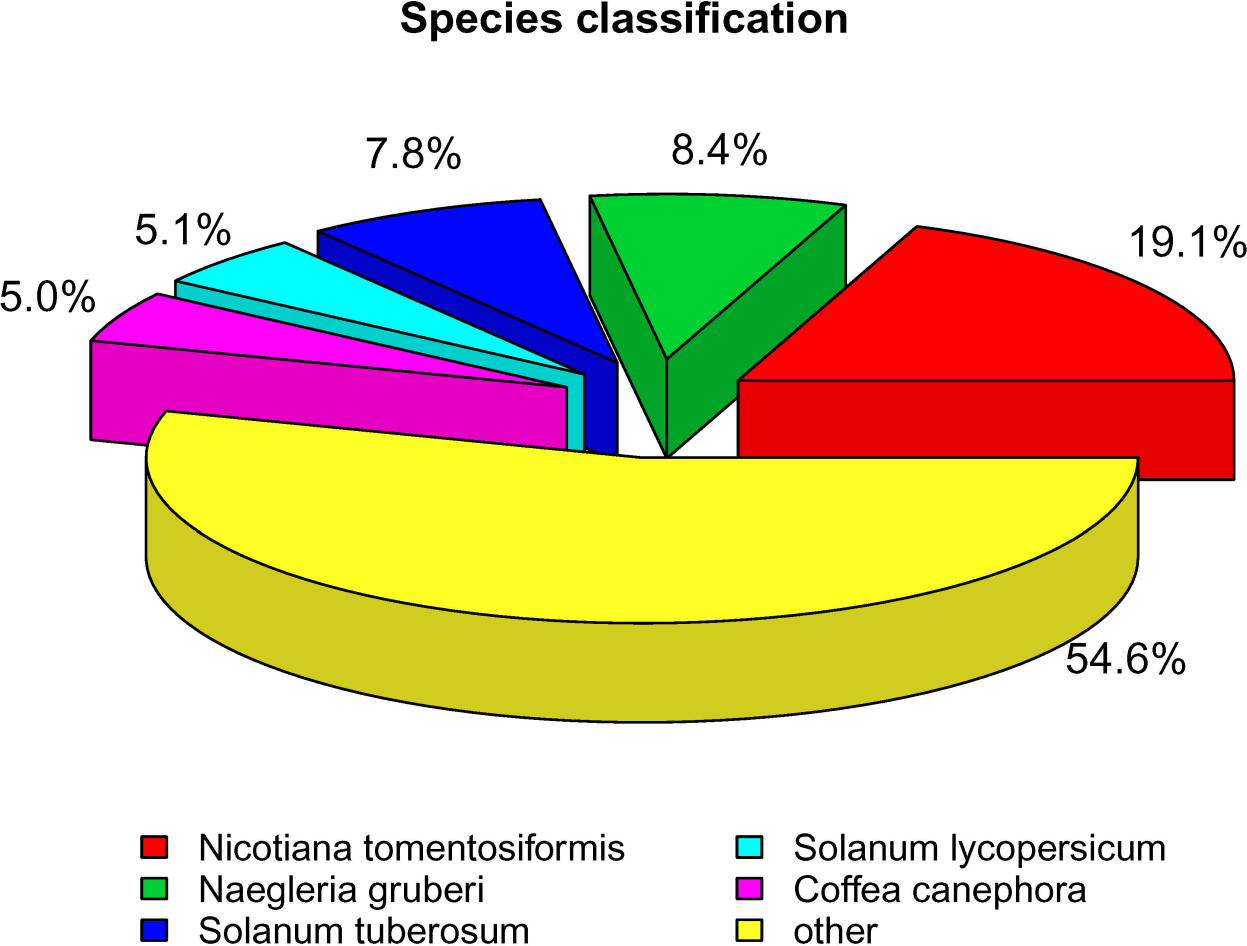
Species distribution of the BLASTX matches of the transcriptome unigenes. This figure shows the species distribution of the unigene BLASTX matches against the NR protein database (cutoff E-value of E<10^−5^) and the proportions of each species.

To annotate the obtained unigenes, we performed a BLASTX search against the NR NCBI protein databases with a cutoff E-value of 10^−5^ based on sequence similarity. In total, 62,605 unigenes were detected (Table 4) that had good comparability with known gene sequences in at least one database, corresponding to approximately 61.38% of the total unigenes; 8,683 (8.51%) unigenes were annotated in all databases, including NR, NCBI non-redundant nucleotide sequences (NT), KO, Swiss-Prot, Protein Family (PFAM), Gene Ontology (GO) and Eukaryotic Ortholog Groups (KOG). As shown in Fig 2, the species that provided the best BLASTX matches (first hit) was *Nicotiana tomentosiformis* (19.1%), with more than 10,000 genes having high homology, followed by *Solanum lycopersicum*, *Naegleria gruberi*, *Coffea canephora* and *Solanum tuberosum*. However, more than half of the unigenes matched other species.

**Table 4:** Statistics of unigenesannotated in public databases.

Search item	Number of unigenes	Percentage (%)
Annotated in NR	56,362	55.26
Annotated in NT	32,361	31.73
Annotated in KO	19,578	19.19
Annotated in Swiss-Prot	38,491	37.74
Annotated in PFAM	38,592	37.83
Annotated in GO	40,918	40.12
Annotated in KOG	22,613	22.17
Annotated in all databases	8,683	8.51
Annotated in at least one database	62,605	61.38
Total unigenes	101,988	100

### GO and KOG classifications

**Fig 3:**
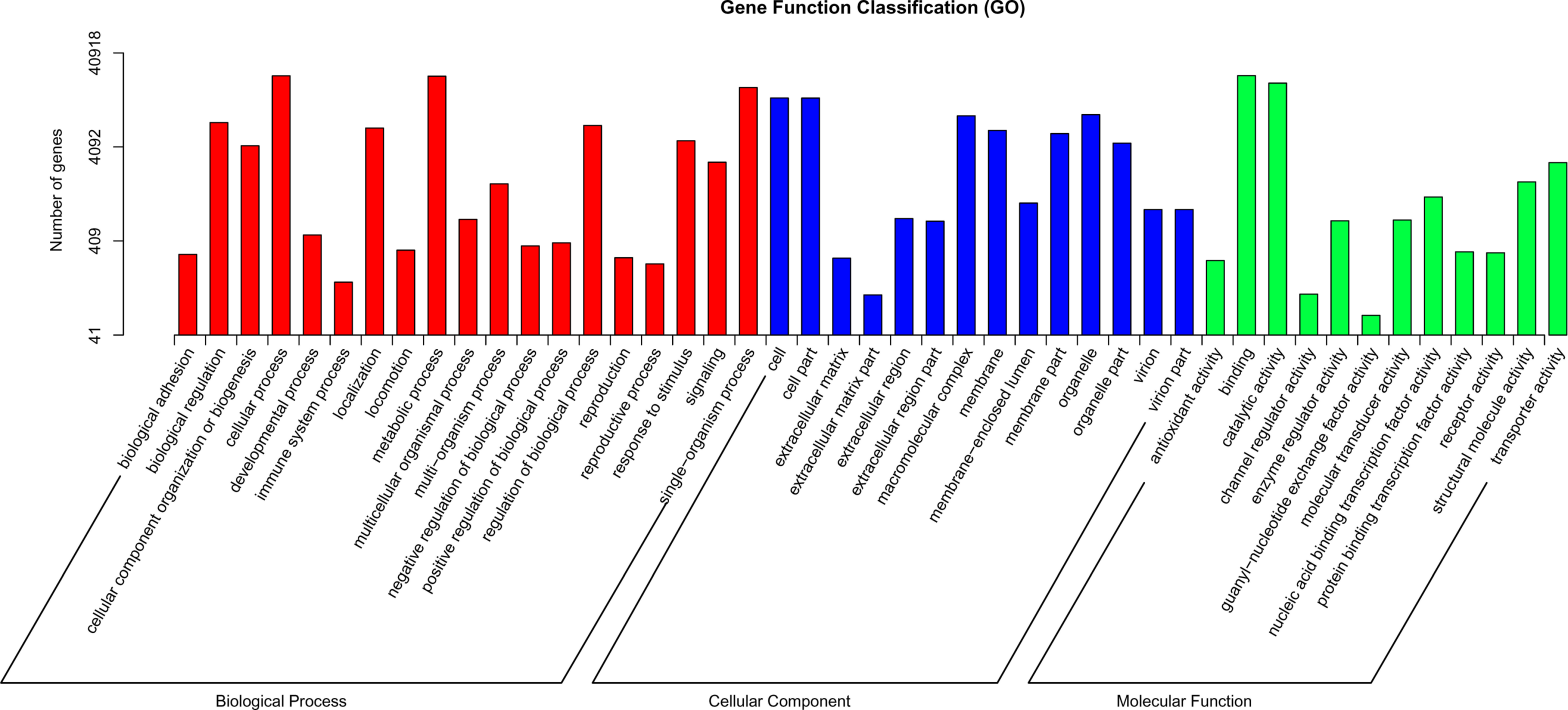
Histogram of GO classifications. The percentages indicate the proportion of unigenes with the GO annotations.

GO classifications offer an ontology of defined terms representing the properties of gene products [18]. According to the results of a BLASTX search against the NR protein database, 40,918 genes (40.1%) were assigned to at least one GO term and were categorized into 46 classes. To better review the GO classifications involving these genes, each GO term was further clustered into its parent term. Ultimately, the classes were clustered into 45 functional groups and three major classifications (Fig 3), and the term “binding” (GO: 0005488) in molecular function represented the largest group (23,444 unigenes), followed by “cellular process” (GO: 0009987) in biological process (23,339 unigenes), “metabolic process” (GO: 0008152) in biological process (23,146 unigenes), and “catalytic” (GO: 0003824) in molecular function (19,536). The smallest groups included the terms “hormone secretion” (GO: 0046879) in biological process (2), “symplast” (GO: 0055044) in cellular component (5), “nucleoid” (GO: 0009295) in cellular component (7) and “synapse” (GO: 0045202) in cellular component (11) (Fig 3).

**Fig 4:**
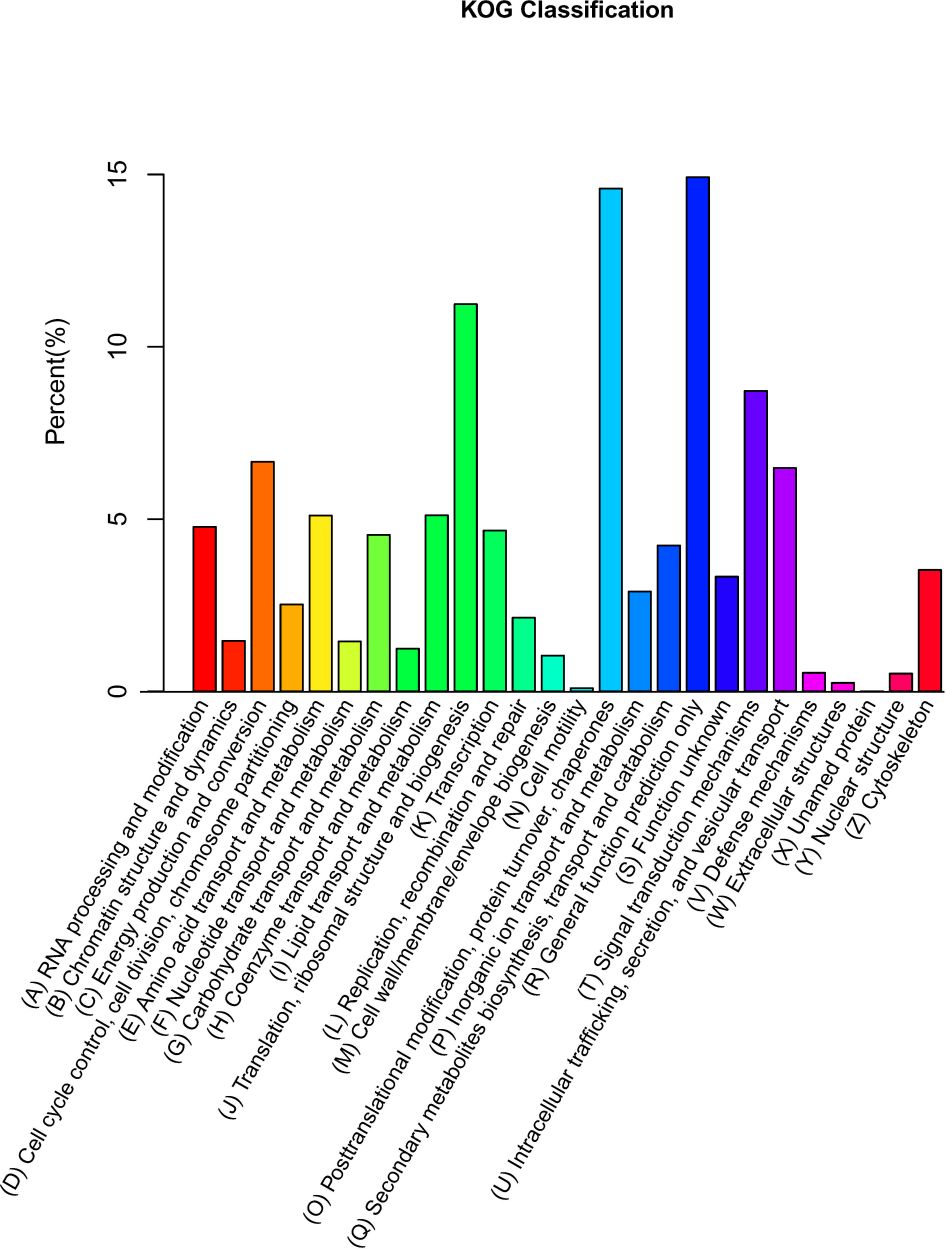
Histogram of the COG classification in sweet potato. The horizontal axis represents the names of the COGs, and the vertical axis represents the proportion of the group of the total number. In total, 22,613 sequences had COG classifications across 25 categories.

A phylogenetic classification was performed using COG databases to classify the potential functions. In total, 22,613 genes were aligned to 26 functional classes (Fig 4). The two largest cluster groups were “general function prediction only” (3,374, 14.9%) and “posttranslational modification, protein turnover, chaperones” (3,299, 14.6%). Interestingly, 122 genes were aligned to the “defense mechanisms” cluster, which is helpful for understanding the mechanisms of resistance to Fusarium wilt in sweet potato.

### KEGG pathway mapping

**Fig 5:**
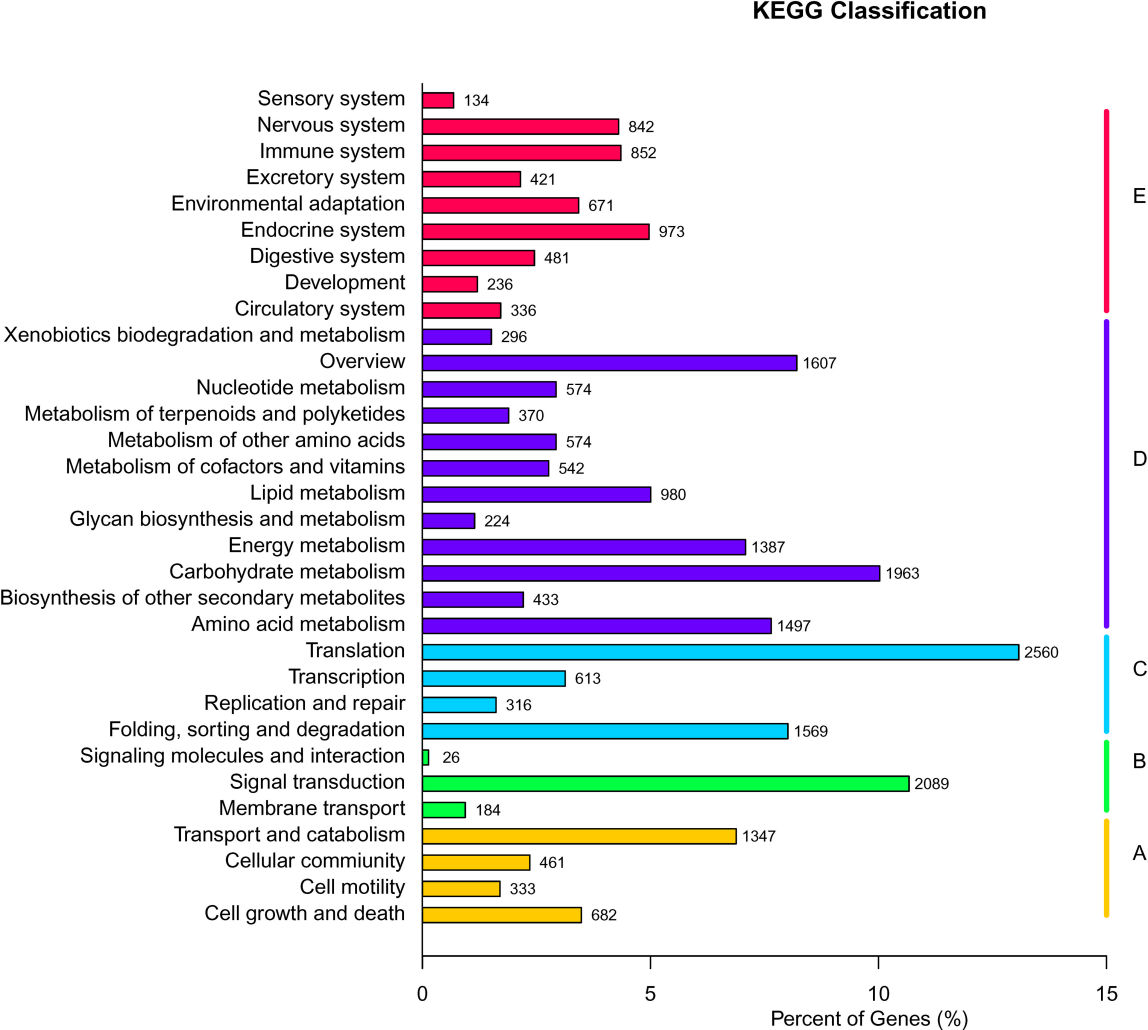
Histogram of KEGG clusters in sweet potato.

The KEGG database is widely used as a reference database of pathway networks for the integration and interpretation of large-scale datasets generated using high-throughput sequencing technology and is helpful for obtaining a better understanding of the functions of genes that are up- or down-regulated in response to the Fusarium wilt pathogen [18]. A BLASTX search against the KEGG protein database was performed using a cutoff E-value of e-5. In total, 19,578 distinct gene sequences were assigned to 276 KEGG pathways. The pathways most represented by unique sequences included translation (2,560 genes), signal transduction (2,089 genes) and carbohydrate metabolism (1,963 genes) (Fig 5). The KEGG annotations reveal the specific enrichment of pathways and the mechanisms involved in the response to Fob infection in sweet potato.

### Gene expression profile of sweet potato in response to Fob

The library was prepared using 3 μg of RNA from each sample, and sequencing was performed on an Illumina HiSeq 2500 platform. The number of unambiguous tags in each library was normalized to the expected number of fragments per kilobase of transcript per million base pairs sequenced (FPKM), and the differentially expressed genes were then compared between different libraries. The DESeq R package (1.10.1) was used to analyze the differential expression between two groups using samples with three biological replicates [32]. DESeq provides a statistical approach for determining differential expression of digital gene expression data using a model based on the negative binomial distribution. Genes with an adjusted P-value <0.05 were considered differentially expressed.

A comparison of the differentially expressed genes between the libraries revealed that many genes were up- or down-regulated in both sweet potato cultivars after inoculation with Fob. Notably, the number of up-regulated genes was higher than the number of down-regulated genes. In the JS57 cultivar, the expression of 6,454 genes was affected by F07 inoculation (JS57-F07 library); of these genes, 3,843 and 2,611 were up-regulated and down-regulated, respectively, compared with the control (JS57-CK library). In the XZH cultivar, the expression of 6,826 genes was affected by inoculation with F07 (XZH-F07 library); of these genes, 5,552 and 1,274 genes were up-regulated and down-regulated, respectively, compared with the control (XZH-CK).

### Experimental validation by qRT-PCR

**Fig 6:**
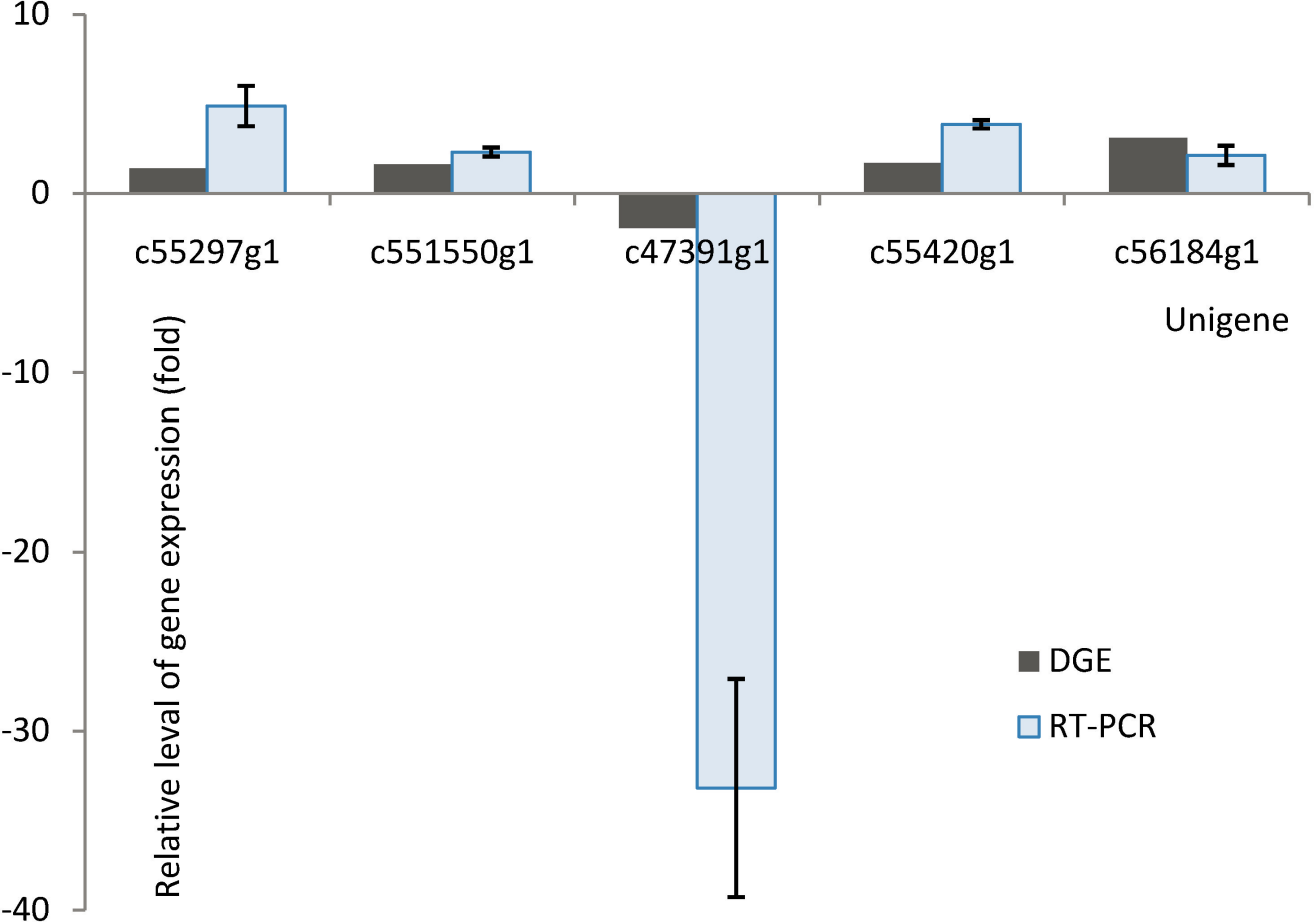
qRT-PCR validation of the Fob-induced fold-changes detected using RNA-Seq. Standard error bars are shown for the fold-changes determined by qRT-PCR.

To confirm the reliability of the Illumina Solexa sequencing data and the differential DGE results, five unigenes were selected for quantitative RT-PCR assays: c47391-g1, c51550-g2, c52297-g1, c54420-g2 and c56184-g1. The results revealed the same expression tendency observed in the DGE analysis in both cultivars (Fig 6). qRT-PCR indicated that c52297-g1, c51550-g2, c54420-g2 and c56184-g1 were up-regulated by 4.88-, 2.30-, 3.85- and 2.12-fold after infection with Fob, respectively, whereas these were found to be up-regulation by 1.40-, 1.62-, 1.71- and 3.1-fold according to the DGE results, respectively. The unigene c47391-g1 was down-regulated 33.19-fold according to the qRT-PCR results but by 1.93-fold according to the DGE results.

### Identification of related genes involved in the defense response

We identified a set of differentially expressed genes involved in the defense response by comparing the commonalities and differences in the libraries of the highly resistant and highly susceptible cultivars upon Fob infection. These genes, include chitin elicitor receptor kinase 1 (CERK), mitogen-activated protein kinase (MAPK), WRKY, NAC, MYB, and ethylene-responsive transcription factor (ERF), as well as resistance (R) genes, pathogenesis-related (PR) genes and genes involved in the salicylic acid (SA) and jasmonic acid (JA) signaling pathways, and the total number of defense-related genes and the differentially expressed genes are shown in Table 5.

**Table 5:** Number of defense-related and differentially expressed genes identified in the response to Fob infection.

Related genes	Gene numbers involved	Significant differential expression	Ratio of differentially expressed genes to total (%)
CERK 1	5	2	40.0
MAPK	66	6	9.1
JA signaling	14	2	14.3
SA signaling	25	4	16.0
ERF	40	14	35.0
WRKY	123	8	6.5
NAC	20	2	10.0
MYB	22	1	4.5
R genes	94	3	3.2
PR genes	57	7	12.3

#### CERK 1 genes

CERk1 is a PRR in host plants that plays a crucial role in chitin perception in the immune response against fungal pathogens [33]. We identified two differentially expressed CERK1 genes (Table 6): c60044-g1 (putative CERK1-like) and c62516-g1 (putative CERK1). c60044-g1 was up-regulated in both JS57-F07 and XZH-F07. Interestingly, c62516-g1 was down-regulated in the highly resistant cultivar JS57-F07, suggesting a complicated interaction between sweet potato and Fob.

**Table 6:** Differentially expressed genes involved in the defense response against Fob infection.

	Gene ID	Blast annotation	JS57-F07 vs JS57-CK	XZH-F07 vs XZH-CK
CERK	c60044-g1	CERK1 [*A*. *thaliana*]	3.2	1.4
	c62516-g1	CERK1-like [*N*. *tomentosiformis*]	-4.1	
MAPK	c51382-g1	Wound-induced MAPK [*N*. *glutinosa*]		0.9
	c54598-g1	MAPK 20 isoform X1 [*N*. *tomentosiformis*]		0.7
	c58290-g1	MAPK [*I*. *batatas*]		1.3
	c58750-g5	MAPK 9 [*N*. *tomentosiformis*]	0.8	1.1
	c65078-g2	MAPK 6 isoform X1 [*N*. *tomentosiformis*]	-1.2	
	c66053-g1	Receptor protein kinase [*R*. *communis*]	-1.8	
JA signaling	c43470-g1	Jasmonic acid 2 [*S*. *lycopersicum*]	3.0	
	c52478-g1	Jasmonate O-methyltransferase [*B*. *rapa*]		-0.9
SA signaling	c56489-g2	Salicylic acid-binding protein 2 [*N*. *benthamiana*]	-1.3	
	c57992-g1	Salicylic acid-binding protein 2-like [*N*. *tomentosiformis*]	-3.2	
	c58625-g1	UDP-glycosyltransferase 74F2-like [*N*. *tomentosiformis*]		-2.0
	c65549-g1	UDP-glycosyltransferase 74F2-like [*N*. *tomentosiformis*]	1.3	-0.7
ERF	c33996-g1	Ethylene-responsive-element-binding protein [*S*. *lycopersicum*]	2.6	
	c45744-g1	Ethylene response factor 3 [*S*. *lycopersicum*]		0.8
	c51416-g2	Ethylene response factor 4 [*S*. *lycopersicum*]		1.2
	c52634-g1	Ethylene response factor 2 [*N*. *tomentosiformis*]		1.3
	c53488-g1	Ethylene response factor 13 [*A*. *thaliana*]	1.2	0.9
	c55705-g1	Ethylene response 2 [*A*. *thaliana*]	2.2	
	c57791-g1	Ethylene-responsive factor [*C*. *chinense*]	3.6	0.6
	c58358-g1	Ethylene response factor 13 [*C*. *arabica*]	1.9	
	c58425-g1	Ethylene receptor homolog [*N*. *tabacum*]	2.0	
	c60985-g1	Ethylene-responsive transcription factor ERF027 [*A*. *thaliana*]		1.6
	c61066-g1	Ethylene-responsive-element-binding factor 5 [*P*. *hybrida*]		-1.0
	c61066-g2	Ethylene response factor 14 [*A*. *deliciosa*]	2.6	
	c64410-g1	Ethylene response factor 1 [*I*. *batatas*]		0.7
	c65518-g1	Ethylene-insensitive protein 3 [*N*. *tabacum*]	0.7	0.5
WRKY	c54420-g2	WRKY61 [*N*. *tomentosiformis*]		1.7
	c56135-g1	WRKY75 [*N*. *tomentosiformis*]		1.6
	c57247-g1	WRKY22 [*C*. *roseus*]	1.8	1.6
	c60589-g1	WRKY71 [*A*. *thaliana*]		1.3
	c64728-g1	WRKY6 [*A*. *thaliana*]		1.1
	c46250-g1	WRKY56 [*A*. *thaliana*]		-2.1
	c50623-g1	WRKY48-like [*S*. *tuberosum*]	-1.4	
	c33599-g2	Protein containing a WRKY-binding domain	3.4	
NAC	c51550-g2	NAC29 [*A*. *thaliana*]		2.1
	c52292-g3	NAC7-like [*M*. *acuminata*]	-3.7	
MYB	c5221-g1	*Ipomoea batatas* IbMYB1-2b	4.6	
PR protein	c35430-g1	PR-1 type-like [*P*. *dactylifera*]		2.1
	c40413-g1	PR class 4 [*Prunus dulcis*]	3.5	
	c42566-g1	PR-4 [*P*. *mume*]	3.2	
	c47344-g1	PR-4A-like isoform X1 [*C*. *arietinum*]	-1.5	
	c54564-g1	PR-1 [*N*. *tomentosiformis*]	-1.5	
	c55964-g1	PR-10 [*N*. *benthamiana*]	5.9	2.6
	c58341-g1	PR-1 type-like [*P*. *dactylifera*]	0.8	
R gene	c56584-g1	NBS-coding resistance protein [*A*. *thaliana*]	9.8	
	c66373-g2	Late blight resistance protein homolog R1B-14 [*N*. *tomentosiformi*s]	-1.8	
	c56383-g1	Resistance gene analog [*I*. *batatas*]		2.4

Note: Significant differences (FDR≤0.001) in relative levels and |log2.Fold-change|≥1 are shown in boldface. FDR, false discovery rate. log2.Fold-change, log2-fold-changes using the ratio base 2 logarithm.INF, the adjusted read count of the gene was 0 in the control sample.

#### MAPK signaling genes

MAPK signaling cascades comprise an MAP kinase kinase kinase (MPKKK), an MAP kinase kinase (MPKK or MAP2K), and an MAP kinase(MPK) that are transduced from the receptor to downstream targets as a signal to sequentially activate the expression of defense-related genes [34]. We identified 66 MAPK-related genes, of which six were differentially expressed. MAPKs have attracted much attention because these genes are involved in the induction and coordination of hormone-dependent and hormone-independent plant responses against necrotrophic fungal pathogens [34].

#### Genes associated with the SA and JA signaling pathways

The plant hormones SA and JA are major defensive players in the regulation of signaling networks underlying resistance to pathogens [35]. SA plays an extensive signaling role in plants, particularly in the defense against biotrophic/hemibiotrophic pathogens, by inducing cell death, activating the expression of defense-related genes, and provoking systemic acquired resistance (SAR) [36, 37]. We identified 20 genes that were associated with the SA pathway (Table 5), and four of these genes were differentially expressed. Two unigenes, c21763-g1 and c39777-g1, were up-regulated in XZH-F07, and both were annotated as S-adenosyl-L-methioninesalicylic acid carboxyl methyltransferase (SAMT), which has been implicated in disease resistance responses in rice [38]. The unigene c58625-g1 was down-regulated in XZH-F07 and annotated as UDP-glycosyltransferase (UDPG), which might influence the accumulation of free SA, leading to changes in the resistance of the host plant [39].

JA-dependent signaling is effective against necrotrophic pathogens [35], and we identified two differentially expressed JA metabolism-related genes that were annotated as jasmonic acid 2 and jasmonate O-methyltransferase. These genes might play an important role in defense against the necrotrophic phase of Fob infection.

#### WRKY transcription factor genes

In total, 123 WRKY genes were identified. Of these 123 WRKY genes, ten (8.1%) were significantly differentially expressed in at least one comparison (Tables 4 and 5). The WRKY transcription factors belong to a large family of transcriptional regulators that are almost exclusively expressed in plants. These transcription factors have diverse biological functions and play important roles in the defense response and hormone-controlled processes by acting as transcriptional activators or repressors that regulate major changes in plants during the early phases of root colonization with fungi [40–42].

Five unigenes (c54420-g2, c56135-g1, c57247-g1, c60589-g1, and c64728-g1) were exclusively up-regulated in XZH-F07 and were identified as putative WRKY61, WRKY75, WRKY22, WRKY71 and WRKY6, respectively, all of which are related to the defense response. In *Arabidopsis*, the WRKY61 gene plays a role in reducing the symptom severity of turnip crinkle virus disease and is associated with plant immunity pathways [43]. WRKY 75 plays a role in decreasing the severity of bacterial soft rot disease and activates a subset of defense-related genes in Chinese cabbage, similar to *Arabidopsis* [44]. WRKY22, WRKY71 and WRKY6 are all involved in rice defense responses. WRKY22 also plays a role in the resistance response to blast [45], and WRKY 71 functions as a transcriptional regulator upstream of OsNPR1 and OsPR1b in defense signaling pathways [46]. WRKY6 positively regulates defense responses by increasing SA concentrations through activation of the SA biosynthesis-related gene OsICS1 [47]. The unigene c46250-g1 was annotated as putative WRKY56 and was exclusively down-regulated in XZH-F07; however, the function of the WRKY56 gene remains unclear. The unigene c50623-g1 was annotated as a putative WRKY48-like gene, and its expression level was significantly lower in JS57-CK than in XZH-CK. WRKY48 was identified as a negative regulator of PR gene expression and basal resistance against the bacterial pathogen *P*. *syringae* in *Arabidopsis* [48]. The unigene c33599-g2 was annotated as a putative WRKY gene because its coding protein contains a WRKY-binding domain, and the expression level of WRKY in JS57-F07 was 3.4-fold higher than that in ZXH-F07, indicating an important role in the defense response.

#### NAC transcription factor genes

NAC transcription factors are a large family of transcriptional regulators that play crucial roles in plant immune responses, basal defense, and SAR [49]. We identified 20 NAC transcription factor genes, and only two (10%) of these genes were significantly differentially expressed in at least one comparison (Table 5). The unigene c51550-g2 was annotated AS NAC29, and its expression level increased in XZH-F07. NAC29 plays important roles in senescence processes and responses to salt and drought stresses in wheat [50]; however, there are no studies of the function of this gene in disease resistance. The other unigene, c52292-g3, was annotated as a NAC7-like gene, and its expression level was 3.7-fold lower in JS57-CK than in XZH-CK, indicating that this gene is also a negative regulator of the defense response.

#### ERF genes

ERFs are responsible for generating tolerance to stress in plants. Most ERFs are transcriptional activators, although some act as transcriptional repressors [51]. We identified 40 ERFs, of which 14 were differentially expressed. These were all up-regulated, except forc61066-g1 (putative ERF 5), indicating that most ERFs are activated in response to Fob invasion. The gene c61066-g1 was annotated as ERF5 and was down-regulated in XZH-F07, indicating that this gene may be a negative regulator of disease resistance in sweet potato. This finding is consistent with results in *Arabidopsis* indicating that ERF5 negatively regulates chitin signaling and plant defense against the fungal pathogen *Alternaria brassicicola* [52].

#### MYB transcription factor genes

The transcription factor MYB family of proteins is large and functionally diverse in all eukaryotes and plays crucial roles in the interaction of regulatory networks that control development, metabolism and the response to biotic or abiotic stresses [53]. The unigene c5221-g1 was annotated as IbMYB1 and was expressed at a 4.6-fold higher level in Js57-CK than in XZH-CK, indicating its important potential function in the defense response. In sweet potato, MYB1 (IbMYB1) stimulates the accumulation of anthocyanin pigmentation in the tuberous roots of sweet potato and the leaves of tobacco and arabidopsis [54–56]. IbMYB1 also improves tolerance and enhances secondary metabolism in transgenic potatoes [57].

#### PR genes

PR proteins are induced in plants upon attack by various pathogens and stresses and perform diverse functions, including plant defense, disease resistance, and antifungal activity [58]. PR proteins play important roles in hypersensitive responses and most likely contribute to SAR [59]. We identified 57 PR genes, of which seven were differentially expressed in at least one comparison and were annotated as PR-1-, PR-4- and PR-10-type proteins (Table 5). PR-1-type proteins appear to be hallmarks of activation of the hypersensitive response and SA-dependent SAR pathways [60]. The PR-4 protein is one of the less well-studied of the 17 PR protein groups and is considered an endochitinase with chitinase activity [61] and a bifunctional enzyme with both RNase and DNase activities [62]. We identified three genes that were annotated as PR-4 type proteins, but further studies are required to better understand their functions. The gene c55964-g1 was annotated as PR-10 and was up-regulated 5.9-foldin JS57-F07, which is markedly higher than the 2.6-fold increase in expression observed in XZH-F07, indicating its important function in the defense against Fob in sweet potato and consistent with its function in peanut [63].

#### R genes

According to the gene-for-gene hypothesis, R proteins recognize and interact with effectors and trigger ETI [12]. We identified 94 R genes in the transcriptome, most of which contain a highly conserved domain characterized by an NBS-LRR structure (data not shown). However, only three of these genes were differentially expressed (Table 5). Interestingly, the unigene c56584-g1 was identified as a putative NBS-coding R protein that was up-regulated 9.8-fold in JS57-F07, indicating a crucial role in the defense response.

## Discussion

Sweet potato is an important crop worldwide and represents a non-model plant with a large, complex genome [18]. The lack of a reference genome sequence is a barrier to the identification of candidate genes involved in disease resistance. The application of high-throughput sequencing technology can generate a large amount of transcriptome data, which is helpful for gaining an understanding of the mechanism of resistance and identifying related genes.

In the present study, we first characterized the transcriptome profile of sweet potato challenged with Fob. Illumina sequencing generated 89,944,188 clean, high-quality reads that were assembled into 101,988 unigenes with an average length of 666 bp, and 61.38% of these were functionally annotated in the NR protein database. Undoubtedly, the results of our study provide an overview of the gene expression profile of sweet potato challenged with Fob and offer abundant sequence data for the discovery of candidate genes involved in the resistance to Fusarium wilt. A large proportion of the unigenes (18,574 unigenes, 18.2%) are longer than 1 kb, facilitating the discovery of defense-related genes.

**Fig 7:**
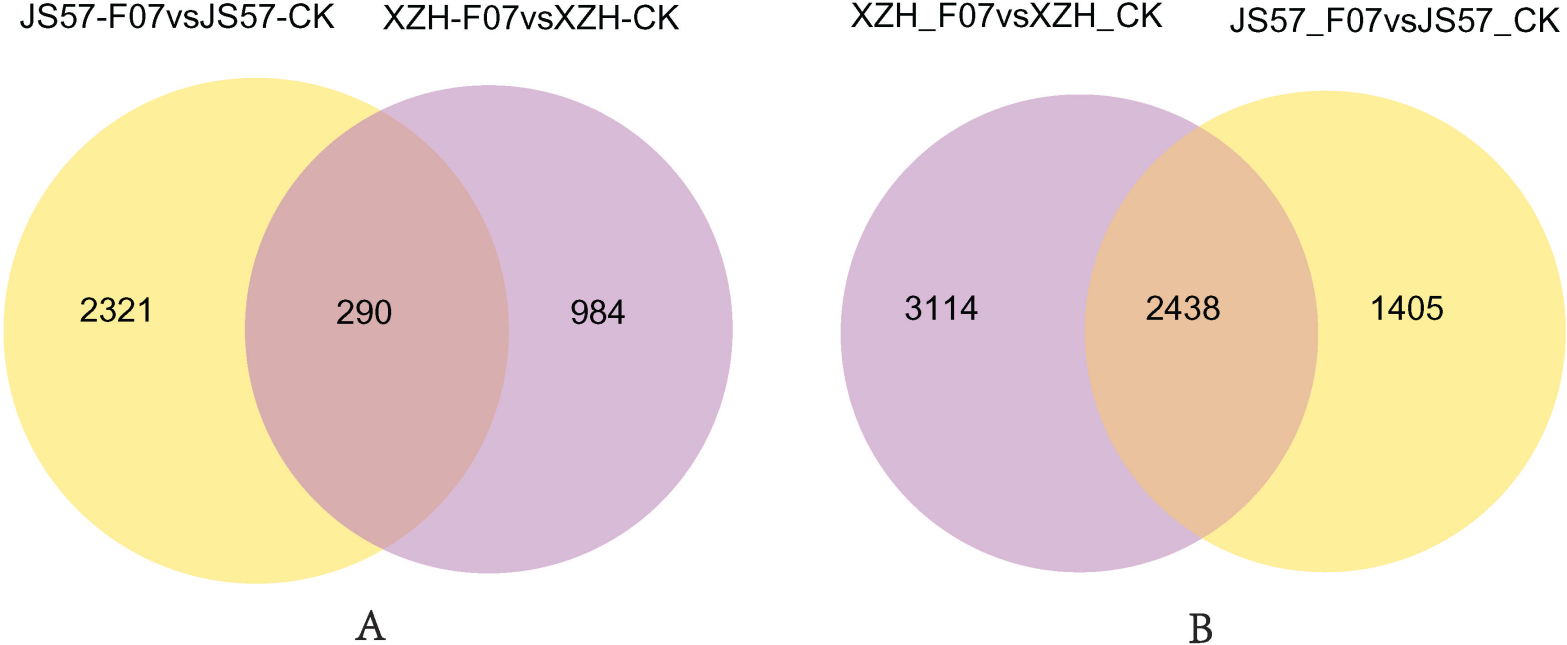
Venn diagram showing overlapping differentially expressed genes in the comparisons of JS57-F07 with JS57-CK and XZH-F07 with XZH-CK.

Many genes were up- and down-regulated in sweet potato when challenged with Fob. A Venn diagram illustrating the overlap in the differentially expressed genes in the comparisons of JS57-F07 with JS57-CK and XZH-F07 with XZH-CK is shown in Fig 7. There were more up-regulated than down-regulated genes, with 5,552 up-regulated and 1,274 down-regulated genes in XZH and 3,843 up-regulated and 2,611 down-regulated genes in JS57. In contrast, in banana infected with Foc TR4, the numbers of up-regulated and down-regulated genes were almost the same [64]. In the two comparisons, 2,438 overlapping differentially expressed up-regulated genes were identified, accounting for 43.91% (2438/5552) and 63.44% (2438/3843) in each comparison. However, only 290 differentially expressed down-regulated genes were detected, accounting for 11.10% (290/2611) and 22.76% (290/1274) in each comparison.

Plants thrive in certain environments and are continuously challenged by various forms of biotic stresses; thus, plants have evolved sophisticated mechanisms to sense biotic aggressors, activate complex phytohormones signaling networks, stimulate plant immune system responses, and regulate the expression of defense-related genes [34]. We identified a set of defense-related genes, including transcription factors, PR genes, R genes, and genes involved in the SA and JA signaling pathways, in the transcriptome.

PAMPs of fungal pathogens, such as chitin, chitosan and endopolygalacturonase, are perceived by PRRs in host plants and activate PTI [34]. CERK1 is an important PRR that plays a crucial role in chitin perception in *Arabidopsis thaliana* in the immune response against fungal pathogens [33]. We identified two CERK1 genes that were differentially expressed in the examined libraries. Intriguingly, c62516-g1 (putative CERK1-like) was down-regulated 4.1-fold in JS57-F07, indicating that effectors of Fob might suppress the expression of c62516-g1 during colonization, thereby restraining PTI in host plants. However, because JS57 is highly resistant to Fob, this plant must have R genes that recognize the effector and trigger ETI to activate defense responses.

The recognition of pathogens by PRRs in plants triggers MAPK cascades and activates SA, JA or ET signaling pathways, facilitating the mounting of defense responses against invading pathogens and inducing the expression of numerous defense-related genes [34]. SA plays a crucial role in plant-pathogen interactions by activating defense responses and is also involved in inducing SAR [65]. SA activates plant immune responses against biotrophic pathogens, whereas JA activates plant immune responses against necrotrophic pathogens [35]. The JA and SA signaling pathways might interact antagonistically [66] or positively [67]. The crosstalk between SA and JA is controlled by a novel function of cytosolic NPR1 [68] and WRKY transcription factors [69]. As a hemibiotrophic pathogen, the lifestyle of Fob includes an initial biotrophic phase (host cell remains alive) and a necrotrophic phase (host tissue is killed) [70]. The crosstalk between the JA and SA signaling pathways might be more complicated in sweet potato during Fob invasion, and further research is required to better understand the interactions during signal transduction.

Effectors enable pathogens to overcome PTI in host plants, and these factors can be recognized by the corresponding R genes of the host plants, leading to ETI [11]. R proteins play a pivotal role in inducing ETI responses. Most R genes encode NB-LRR proteins [11]. We identified 94 R genes in the transcriptome, and three of these genes were differentially expressed. Interestingly, c56584-g1 (putative NBS-coding resistance protein) was up-regulated 9.8-fold in JS57-F07, indicating its important role in defense responses against Fob. Cloning of the R gene from sweet potato and the effector gene from Fob and further studies of the interaction between these genes are required to elucidate the defense mechanism of sweet potato.

## Conclusions

In the present study, we de novo constructed and characterized the transcriptomes of sweet potato challenged with Fob and identified 101,988 unigenes, generating a broad survey of genes involved in disease resistance. We performed DGE profiling analyses of sweet potato challenged with Fob and calculated the numbers of differentially expressed genes between libraries, identifying several up- and down-regulated genes. A set of candidate genes in sweet potato involved in the disease response to Fusarium wilt were identified, including WRKY and NAC transcription factors, R genes, PR genes and SA pathway-related genes. Some of these genes, such as c56584-g1 (putative R gene) and c55964-g1 (putative PR-10), were significantly up-regulated and implicated in the defense response against Fob. These data provide an understanding of the molecular mechanisms of disease resistance. This sequence resource will be valuable for genetic and genomic studies and will accelerate breeding programs for sweet potato with Fusarium wilt resistance.

## Abbreviations

CERK1: Chitinelicitor receptor kinase 1
MAPK: Mitogen-activated protein kinase
PR: Pathogenesis related
SA: Salicylic acid
ET: Ethylene
JA: Jasmonic acid
DGE: Digital gene expression
ETI: Effector-triggered immunity
Fob: *Fusarium oxysporum f*. *sp*. *batatas*
GO: Gene Ontology
KEGG: Encyclopedia of Genes and Genomes pathway database
PAMPs: Pathogen-associated molecular patterns
PRRs: Pattern recognition receptors
PTI: PAMP-triggered immunity
SAR: Systemic acquired resistance
F07: Strain of *Fusarium oxysporum* f. sp. *batatas* with strong pathogenicity
JS57: JinShan 57 (sweet potato cultivar with high resistance to Fusarium wilt disease)
JS57-F07: Sweet potato cultivar JS57 inoculated with F07
XZH: XinZhongHua (sweet potato cultivar with high susceptibility to Fusarium wilt disease)
XZH-F07: Sweet potato cultivar XZH inoculated with F07

## References

[1] Afuape SO, Nwankwo IIM, Omodamiro RM, Echendu TNC, Toure A. Studies on some important consumer and processing traits for breeding sweet potato for varied end-uses. American Journal of Experimental Agriculture. 2014;4(1):114–24. doi: 10.9734/AJEA/2014/5827

[2] FAOSTAT. Food and Agricultural Organization of the United Nations Crop Production Figures for Sweetpotato, released by FAO Statistics Division, June,18, 2016. Food and agriculture organization of the united nations statistics division. 2014.

[3] Fravel D, Olivain C, Alabouvette C. Fusarium oxysporum and its biocontrol. New Phytologist. 2003;157(3):10 doi: 10.1046/j.1469-8137.2003.00700.x 10.1046/j.1469-8137.2003.00700.x 33873407

[4] Fang S, Chen Y, Guo X. Identifying and screening sweet potato varieties resistance to bacterial wilt and fusarium wilt diseases. Journal of Plant Genetic Resources. 2001;2(1):37–9.

[5] Clark CA, Hyun JW, Hoy MW. Relationships among wilt-inducing isolates of Fusarium oxysporum from sweetpotato and tobacco. Plant Disease. 1998;82(5):530–6. doi: 10.1094/PDIS.1998.82.5.530 10.1094/PDIS.1998.82.5.530 30856983

[6] Fang S, Chen F, Xu M, Lin W. A preliminary study on inheritance of fusarium wilt resistance of sweet potato cultivars. Journal of Fujian Agricultural University. 1997;26(4):446–8.

[7] Lei J, Yang XS, Guo WW, Wen-Jin SU, Wang LJ. Advances in Research on Sweet Potato Fusarium Wilt. Hubei Agricultural Sciences. 2011;50(23):4775–7.

[8] Pei Y, Zeng F, Peng J, Long H, Guo J. Advances in Fusarium Wilt Disease and the Mechanism of Interaction Between Fusarium and Its Host. Journal of Tropical Biology. 2014;5(1):92–100.

[9] Yin X, Xu B, Zheng W, Zeng H, Ma W, Wang J, et al Histological observation of banana root infected by Fusarium oxysporum f.sp.cubense. Acta Phytopathologica Sinica. 2011;41(6):570–5.

[10] Stahl EA, Bishop JG. Plant-pathogen arms races at the molecular level. Current Opinion in Plant Biology. 2000;3(4):299–304. 10873849 10.1016/s1369-5266(00)00083-2

[11] Jones JD, Dangl JL. The plant immune system. Nature. 2006;444(7117):323–9. doi: 10.1038/nature05286 17108957 10.1038/nature05286

[12] Thatcher LF, Gardiner DM, Kazan K, Manners JM. A highly conserved effector in Fusarium oxysporum is required for full virulence on Arabidopsis. Molecular Plant-Microbe Interactions. 2011;25(2):180–90. doi: 10.1094/MPMI-08-11-0212 21942452 10.1094/MPMI-08-11-0212

[13] Frank T, Martijn R. The arms race between tomato and Fusarium oxysporum. Molecular Plant Pathology. 2010;11(2):309–14. doi: 10.1111/j.1364-3703.2009.00605.x 20447279 10.1111/j.1364-3703.2009.00605.x PMC6640361

[14] Saucet SB, Ma Y, Sarris PF, Furzer OJ, Sohn KH, Jones JD. Two linked pairs of Arabidopsis TNL resistance genes independently confer recognition of bacterial effector AvrRps4. Nature Communications. 2015;6 doi: 10.1038/ncomms7338 25744164 10.1038/ncomms7338

[15] Imam J, Alam S, Mandal NP, Variar M, Shukla P. Molecular screening for identification of blast resistance genes in North East and Eastern Indian rice germplasm (Oryza sativa L.) with PCR based makers. Euphytica. 2014;196(2):199–211. doi: 10.1007/s10681-013-1024-x

[16] Parmar P, Subramanian RB. Isolation of NBS-LRR class resistant gene (I2 gene) from tomato cultivar Heamsona. 2013;16: 6076–8. doi: 10.5897/AJB2013.12194

[17] Tameling WI, Elzinga SD, Darmin PS, Vossen JH, Takken FL, Haring MA, et al The tomato R gene products I-2 and MI-1 are functional ATP binding proteins with ATPase activity. Plant Cell. 2002;14(11):2929–39. doi: 10.1105/tpc.005793 12417711 10.1105/tpc.005793 PMC152737

[18] Xie F, Burklew CE, Yang Y, Liu M, Xiao P, Zhang B, et al De novo sequencing and a comprehensive analysis of purple sweet potato (Impomoea batatas L.) transcriptome. Planta. 2012;236(1):101–13. doi: 10.1007/s00425-012-1591-4 22270559 10.1007/s00425-012-1591-4

[19] Xiao J, Jin X, Jia X, Wang H, Cao A, Zhao W, et al Transcriptome-based discovery of pathways and genes related to resistance against Fusarium head blight in wheat landrace Wangshuibai. Bmc Genomics. 2013;14(1):1–19. doi: 10.1186/1471-2164-14-197 23514540 10.1186/1471-2164-14-197 PMC3616903

[20] Li C, Shao J, Wang Y, Li W, Guo D, Yan B, et al Analysis of banana transcriptome and global gene expression profiles in banana roots in response to infection by race 1 and tropical race 4 of Fusarium oxysporum f. sp. cubense. Bmc Genomics. 2013;14(6):1–16. doi: 10.1186/1471-2164-14-851 24304681 10.1186/1471-2164-14-851 PMC4046742

[21] Firon N, Labonte D, Villordon A, Kfir Y, Solis J, Lapis E, et al Transcriptional profiling of sweetpotato (Ipomoea batatas) roots indicates down-regulation of lignin biosynthesis and up-regulation of starch biosynthesis at an early stage of storage root formation. Bmc Genomics. 2013;14(1):1–25. doi: 10.1186/1471-2164-14-460 23834507 10.1186/1471-2164-14-460 PMC3716973

[22] Roland S, Luz RT, Omar P, Genoveva R, Ronald FR, Rocio A, et al A sweetpotato gene index established by de novo assembly of pyrosequencing and Sanger sequences and mining for gene-based microsatellite markers. BMC Genomics. 2010;11:604 doi: 10.1186/1471-2164-11-604 20977749 10.1186/1471-2164-11-604 PMC3017860

[23] Wang Z, Li J, Luo Z, Huang L, Chen X, Fang B, et al Characterization and development of EST-derived SSR markers in cultivated sweetpotato (Ipomoea batatas). Bmc Plant Biology. 2011;11(5):1–9. doi: 10.1186/1471-2229-11-139 22011271 10.1186/1471-2229-11-139 PMC3206431

[24] Wang Z, Fang B, Chen X, Liao M, Chen J, Zhang X, et al Temporal patterns of gene expression associated with tuberous root formation and development in sweetpotato (Ipomoea batatas). Bmc Plant Biology. 2015;15(1):1–13. doi: 10.1186/s12870-015-0567-5 26174091 10.1186/s12870-015-0567-5 PMC4502468

[25] Haas BJ, Papanicolaou A, Yassour M, Grabherr M, Blood PD, Bowden J, et al De novo transcript sequence reconstruction from RNA-Seq: reference generation and analysis with Trinity. Nature Protocols. 2013;8(8):1494–512. doi: 10.1038/nprot.2013.084 23845962 10.1038/nprot.2013.084 PMC3875132

[26] Grabherr MG, Haas BJ, Yassour M, Levin JZ, Thompson DA, Amit I, et al Full-length transcriptome assembly from RNA-Seq data without a reference genome. Nature Biotechnology. 2011;29(7):644–52. doi: 10.1038/nbt.1883 21572440 10.1038/nbt.1883 PMC3571712

[27] Götz S, Garcíagómez JM, Terol J, Williams TD, Nagaraj SH, Nueda MJ, et al High-throughput functional annotation and data mining with the Blast2GO suite. Nucleic Acids Research. 2008;36(10):3420–35. doi: 10.1093/nar/gkn176 18445632 10.1093/nar/gkn176 PMC2425479

[28] Park SC, Kim YH, Ji CY, Park S, Jeong JC, Lee HS, et al Stable Internal Reference Genes for the Normalization of Real-Time PCR in Different Sweetpotato Cultivars Subjected to Abiotic Stress Conditions. Plos One. 2012;7(12). doi: 10.1371/journal.pone.0051502 23251557 10.1371/journal.pone.0051502 PMC3520839

[29] Grabherr MG, Haas BJ, Yassour M, Levin JZ, Thompson DA, Amit I, et al Trinity: reconstructing a full-length transcriptome without a genome from RNA-Seq data. Nature Biotechnology. 2013;29(7):644–52. doi: 10.1038/nbt.1883 10.1038/nbt.1883 PMC3571712 21572440

[30] Foroud NA, Ouellet T, Laroche A, Oosterveen B, Jordan MC, Ellis BE, et al Differential transcriptome analyses of three wheat genotypes reveal different host response pathways associated with Fusarium head blight and trichothecene resistance. Plant Pathology. 2012;61(2):296–314. doi: 10.1111/j.1365-3059.2011.02512.x

[31] Li R, Zhai H, Kang C, Liu D, He S, Liu Q. De Novo Transcriptome Sequencing of the Orange-Fleshed Sweet Potato and Analysis of Differentially Expressed Genes Related to Carotenoid Biosynthesis. International Journal of Genomics. 2015;2015(13):1–10. doi: 10.1155/2015/843802 26649293 10.1155/2015/843802 PMC4663004

[32] Anders S, Huber W. Differential expression analysis for sequence count data. Genome Biology. 2010;11(10):1–12. 10.1186/gb-2010-11-10-r106 PMC3218662 20979621

[33] Wan J, Zhang XC, Neece D, Ramonell KM, Clough S, Kim SY, et al A LysM Receptor-like Kinase Plays a Critical Role in Chitin Signaling and Fungal Resistance in Arabidopsis. 2008;20(2):471–81. doi: 10.1105/tpc.107.056754 18263776 10.1105/tpc.107.056754 PMC2276435

[34] Pandey D, Gaur M, Sajeesh PK, Kumar A. Plant Defense Signaling and Responses Against Necrotrophic Fungal Pathogens. Journal of Plant Growth Regulation. 2016:1–16. doi: 10.1007/s00344-016-9600-7

[35] Yang YX, Ahammed GJ, Wu C, Fan SY, Zhou YH. Crosstalk among Jasmonate, Salicylate and Ethylene Signaling Pathways in Plant Disease and Immune Responses. Current Protein & Peptide Science. 2015;16(5):450–61. doi: 10.2174/1389203716666150330141638 25824390 10.2174/1389203716666150330141638

[36] Vlot AC, Dempsey DA, Klessig DF. Salicylic Acid, a Multifaceted Hormone to Combat Disease. Annual Review of Phytopathology. 2009;47:177–206. doi: 10.1146/annurev.phyto.050908.135202 19400653 10.1146/annurev.phyto.050908.135202

[37] Lemarié S, Robert-Seilaniantz A, Lariagon C, Lemoine J, Marnet N, Jubault M, et al Both the Jasmonic Acid and the Salicylic Acid Pathways Contribute to Resistance to the Biotrophic Clubroot Agent Plasmodiophora brassicae in Arabidopsis. Plant & Cell Physiology. 2015;56(11). doi: 10.1093/pcp/pcv127 26363358 10.1093/pcp/pcv127

[38] Xu R, Song F, Zheng Z. OsBISAMT1, a gene encoding S-adenosyl-L-methionine:salicylic acid carboxyl methyltransferase, is differentially expressed in rice defense responses. Molecular Biology Reports. 2006;33(3):223–31. doi: 10.1007/s11033-005-4823-x 16850192 10.1007/s11033-005-4823-x

[39] Boachon B, Gamir J, Pastor V, Erb M, Dean JV, Flors V, et al Role of two UDP-Glycosyltransferases from the F group of Arabidopsis in resistance against Pseudomonas syringae. European Journal of Plant Pathology. 2014;139(4):707–20. doi: 10.1007/s10658-014-0424-7

[40] Bakshi M, Oelmüller R. WRKY transcription factors: Jack of many trades in plants. Plant Signaling & Behavior. 2014;9(1):e27700–e. doi: 10.4161/psb.27700 24492469 10.4161/psb.27700 PMC4091213

[41] Jimmy JL, Babu S. Role of OsWRKY transcription factors in rice disease resistance. Tropical Plant Pathology. 2015;40(6):355–61. doi: 10.1007/s40858-015-0058-0

[42] Gallou A, Declerck S, Cranenbrouck S. Transcriptional regulation of defence genes and involvement of the WRKY transcription factor in arbuscular mycorrhizal potato root colonization. Functional & Integrative Genomics. 2012;12(1):183–98. doi: 10.1007/s10142-011-0241-4 21811781 10.1007/s10142-011-0241-4

[43] Gao R, Liu P, Yong Y, Sek-Man W. Genome-wide transcriptomic analysis reveals correlation between higher WRKY61 expression and reduced symptom severity in Turnip crinkle virus infectedArabidopsis thaliana. Scientific Reports. 2016;6 doi: 10.1038/srep24604 27086702 10.1038/srep24604 PMC4834565

[44] Choi C, Park S, Ahn I, Bae S, Hwang DJ. Generation of Chinese cabbage resistant to bacterial soft rot by heterologous expression of Arabidopsis WRKY75. Plant Biotechnology Reports. 2016;10(5):301–7. doi: 10.1007/s11816-016-0406-7

[45] Abbruscato P, Nepusz T, Mizzi L, Corvo MD, Morandini P, Fumasoni I, et al OsWRKY22, a monocot WRKY gene, plays a role in the resistance response to blast. Molecular Plant Pathology. 2012;13(8):828–41. doi: 10.1111/j.1364-3703.2012.00795.x 22443363 10.1111/j.1364-3703.2012.00795.x PMC6638809

[46] Liu X, Bai X, Wang X, Chu C. OsWRKY71, a rice transcription factor, is involved in rice defense response. Journal of Plant Physiology. 2007;164(8):969–79. doi: 10.1016/j.jplph.2006.07.006 16919842 10.1016/j.jplph.2006.07.006

[47] Choi C, Hwang SH, Fang IR, † SIK, Sang RP, Ahn I, et al Molecular characterization of Oryza sativa WRKY6, which binds to W-box-like element 1 of the Oryza sativa pathogenesis-related (PR) 10a promoter and confers reduced susceptibility to pathogens. New Phytologist. 2015;208(3):846–59. doi: 10.1111/nph.13516 26083148 10.1111/nph.13516

[48] Xing DH, Lai ZB, Zheng ZY, Vinod KM, Fan BF, Chen ZX. Stress- and Pathogen-Induced Arabidopsis WRKY48 is a Transcriptional Activator that Represses Plant Basal Defense. Molecular Plant. 2008;1(3):459–70. doi: 10.1093/mp/ssn020 19825553 10.1093/mp/ssn020

[49] Nuruzzaman M, Sharoni AM, Kikuchi S. Roles of NAC transcription factors in the regulation of biotic and abiotic stress responses in plants. Frontiers in Microbiology. 2013;4(4):248 doi: 10.3389/fmicb.2013.00248 24058359 10.3389/fmicb.2013.00248 PMC3759801

[50] Huang Q, Wang Y, Li B, Chang J, Chen M, Li K, et al TaNAC29, an NAC transcription factor from wheat, enhances salt and drought tolerance in transgenic Arabidopsis. BMC Plant Biology. 2015;15(1):1–15. doi: 10.1186/s12870-015-0644-9 26536863 10.1186/s12870-015-0644-9 PMC4632686

[51] Kamsvågmagnusson T, Thorsellcederberg J, Svanberg A, Von EL, Arvidson J, Mellgren K, et al Role of Ethylene Response Transcription Factor (ERF) and Its Regulation in Response to Stress Encountered by Plants. Plant Molecular Biology Reporter. 2014;33(3):1–11. doi: 10.1007/s11105-014-0799-9

[52] Son GH, Wan J, Kim HJ, Nguyen XC, Chung WS, Hong JC, et al Ethylene-responsive element-binding factor 5, ERF5, is involved in chitin-induced innate immunity response. Molecular Plant-Microbe Interactions. 2012;25(1):48–60. doi: Ethylene-responsive element-binding factor 5, ERF5, is involved in chitin-induced innate immunity response. doi: 10.1094/MPMI-06-11-0165 21936663 10.1094/MPMI-06-11-0165

[53] Dubos C, Stracke R, Grotewold E, Weisshaar B, Martin C, Lepiniec L. MYB transcription factors in Arabidopsis. Trends in Plant Science. 2010;15(10):573–81. doi: 10.1016/j.tplants.2010.06.005 20674465 10.1016/j.tplants.2010.06.005

[54] An CH, Lee KW, Lee SH, Jeong YJ, Woo SG, Chun H, et al Heterologous expression of IbMYB1a by different promoters exhibits different patterns of anthocyanin accumulation in tobacco. Plant Physiology & Biochemistry. 2015;89:1–10. doi: 10.1016/j.plaphy.2015.02.002 25681576 10.1016/j.plaphy.2015.02.002

[55] Chu H, Jeong JC, Kim WJ, Chung DM, Jeon HK, Ahn YO, et al Expression of the sweetpotato R2R3-type IbMYB1a gene induces anthocyanin accumulation in Arabidopsis. Physiologia Plantarum. 2013;148(2):189–99. doi: 10.1111/j.1399-3054.2012.01706.x 23039825 10.1111/j.1399-3054.2012.01706.x

[56] Park SC, Kim YH, Kim SH, Jeong YJ, Kim CY, Lee JS, et al Overexpression of the IbMYB1 gene in an orange-fleshed sweet potato cultivar produces a dual-pigmented transgenic sweet potato with improved antioxidant activity. Physiologia Plantarum. 2015;153(4):525–37. doi: 10.1111/ppl.12281 25220246 10.1111/ppl.12281

[57] Cheng YJ, Kim MD, Deng XP, Kwak SS, Chen W. Enhanced salt stress tolerance in transgenic potato plants expressing IbMYB1, a sweet potato transcription factor. Journal of Microbiology & Biotechnology. 2013;23(12):1737–46. doi: 10.4014/jmb.1307.07024 24378636 10.4014/jmb.1307.07024

[58] Singh R, Tiwari JK, Sharma V, Singh BP, Rawat S, Singh R, et al Role of Pathogen related protein families in defence mechanism with potential role in applied biotechnology. International Journal of Advanced Research. 2014;2:210–26.

[59] Loon LCV, Strien EAV. The families of pathogenesis-related proteins, their activities, and comparative analysis of PR-1 type proteins. Physiological & Molecular Plant Pathology. 2002;55(2):85–97. doi: 10.1006/pmpp.1999.0213

[60] Lu S, Faris JD, Sherwood R, Friesen TL, Edwards MC. A dimeric PR‐1‐type pathogenesis‐related protein interacts with ToxA and potentially mediates ToxA‐induced necrosis in sensitive wheat. Molecular Plant Pathology. 2014;15(7):650 doi: 10.1111/mpp.12122 24433289 10.1111/mpp.12122 PMC6638811

[61] Brunner FA, Fritig B, Legrand M. Substrate specificities of tobacco chitinases. Plant Journal. 2010;14(2):225–34. doi: 10.1046/j.1365-313X.1998.00112.x 10.1046/j.1365-313x.1998.00116.x 9628018

[62] Guevaramorato MA, de Lacoba MG, Garcíaluque I, Serra MT. Characterization of a pathogenesis-related protein 4 (PR-4) induced in Capsicum chinense L3 plants with dual RNase and DNase activities. Journal of Experimental Botany. 2010;61(12):3259–71. doi: 10.1093/jxb/erq148 20511278 10.1093/jxb/erq148 PMC2905194

[63] Chadha P, Das RH. A pathogenesis related protein, AhPR10 from peanut: an insight of its mode of antifungal activity. Planta. 2006;225(1):213–22. doi: 10.1007/s00425-006-0344-7 16832688 10.1007/s00425-006-0344-7

[64] Wang Z, Zhang JB, Jia CH, Liu JH, Li YQ, Yin XM, et al De Novo characterization of the banana root transcriptome and analysis of gene expression under Fusarium oxysporum f. sp. Cubense tropical race 4 infection. Bmc Genomics. 2012;13(12):1–15. doi: 10.1186/1471-2164-13-650 23170772 10.1186/1471-2164-13-650 PMC3534498

[65] Gaffney T, Friedrich L, Vernooij B, Negrotto D, Nye G, Uknes S, et al Requirement of salicylic Acid for the induction of systemic acquired resistance. Science. 1993;261(5122):754 doi: 10.1126/science.261.5122.754 17757215 10.1126/science.261.5122.754

[66] Grant M, Lamb C. Systemic immunity. Current Opinion in Plant Biology. 2006;9(4):414–20. doi: 10.1016/j.pbi.2006.05.013 16753329 10.1016/j.pbi.2006.05.013

[67] Tamaoki D, Seo S, Yamada S, Kano A, Miyamoto A, Shishido H, et al Jasmonic acid and salicylic acid activate a common defense system in rice. Plant Signaling & Behavior. 2013;8(6):e24260–e. doi: 10.4161/psb.24260 23518581 10.4161/psb.24260 PMC3906320

[68] Spoel SH, Koornneef A, Claessens SM, Korzelius JP, Van Pelt JA, Mueller MJ, et al NPR1 modulates cross-talk between salicylate- and jasmonate-dependent defense pathways through a novel function in the cytosol. Plant Cell. 2003;15(3):760–70. doi: 10.1105/tpc.009159 12615947 10.1105/tpc.009159 PMC150028

[69] Pieterse CM, Van dDD, Zamioudis C, Leonreyes A, Van Wees SC. Hormonal modulation of plant immunity. Annual Review of Cell & Developmental Biology. 2012;28(1):489–521. doi: 10.1146/annurev-cellbio-092910-154055 22559264 10.1146/annurev-cellbio-092910-154055

[70] Münch S, Lingner U, Floss DS, Ludwig N, Sauer N, Deising HB. The hemibiotrophic lifestyle of Colletotrichum species. Journal of Plant Physiology. 2008;165(1):41–51. doi: 10.1016/j.jplph.2007.06.008 17765357 10.1016/j.jplph.2007.06.008

